# Mechanism of LSD1 in oxygen-glucose deprivation/reoxygenation-induced pyroptosis of retinal ganglion cells via the miR-21-5p/NLRP12 axis

**DOI:** 10.1186/s12868-022-00747-3

**Published:** 2022-11-10

**Authors:** Xiuling Yu, Tongtong Niu, Chi Liu

**Affiliations:** Department of Ophthalmology, China Medical University the Fourth People’s Hospital of Shenyang, No.20 Huang He Street, Huang Gu District, Shenyang City, 110031 Liaoning Province China

**Keywords:** Oxygen-glucose deprivation/reoxygenation, Retinal ganglion cells, LSD1, Histone H3, Pyroptosis, miR-21-5p, NLRP12, Epigenetic modification

## Abstract

**Background:**

Retinal ganglion cells (RGCs) are important retinal neurons that connect visual receptors to the brain, and lysine-specific demethylase 1 (LSD1) is implicated in the development of RGCs. This study expounded the mechanism of LSD1 in oxygen-glucose deprivation/reoxygenation (OGD/R)-induced pyroptosis of RGCs.

**Methods:**

Mouse RGCs underwent OGD/R exposure, and then RGC viability was examined using the cell counting kit-8 method. The mRNA levels of Caspase 1, the protein levels of NOD-like receptor family pyrin domain-containing 3 (NLRP3), N-terminal fragment of gasdermin D (GSDMD-N), and cleaved-Caspase1, and the concentrations of interleukin (IL)-1β and IL-18 were respectively examined. Subsequently, LSD1 expression was intervened to explore the underlying effect of LSD1 on OGD/R-induced pyroptosis of RGCs. Afterwards, the enrichments of LSD1 and histone H3 lysine 4 methylation (H3K4me) 1/2 on the microRNA (miR)-21-5p promoter were determined using chromatin-immunoprecipitation assay. And the binding interaction between miR-21-5p and NLRP12 was detected using dual-luciferase and RNA pull-down assays. Finally, the effects of miR-21-5p/NLRP12 on LSD1-mediated pyroptosis of RGCs were verified through functional rescue experiments.

**Results:**

OGD/R treatment increased pyroptosis of RGCs and LSD1 expression. Silencing LSD1 declined levels of Caspase 1 mRNA, NLRP3, GSDMD-N, cleaved-Caspase1, IL-1β, and IL-18 and limited pyroptosis of OGD/R-treated RGCs. Mechanically, LSD1 suppressed miR-21-5p expression via demethylation of H3K4me2 on the miR-21-5p promoter to hamper the binding of miR-21-5p to NLRP12, and thereby increased NLRP12 expression. Silencing miR-21-5p or overexpressing NLRP12 facilitated OGD/R-induced pyroptosis of RGCs.

**Conclusion:**

LSD1-mediated demethylation of H3K4me2 decreased miR-21-5p expression to increase NLRP12 expression, promoting pyroptosis of OGD/R-treated RGCs.

## Backgrounds

Retinal ganglion cells (RGCs) are defined as the first class of crucial retinal neurons in the retina that transmit visual input from the retina to the visual processing areas of the brain [1]. Dysfunction or injury of RGCs can lead to ocular disorders and conditions, such as glaucoma, diabetic retinopathy, and ischemic or demyelinating optic neuritis [2]. Ischemia/reperfusion (I/R) injury refers to injuries to tissues or organs resulting from the initial interruption of blood flow and the subsequent restoration of blood flow to organs, and it is a major cause of disability and even death [3]. Oxygen-glucose deprivation/reoxygenation (OGD/R) for cells is commonly used to simulate cell I/R injury models [4, 5], but OGD/R cell models are limited to simulating I/R injury and not equivalent to I/R injury. Retinal I/R causes retinal injury, characterized by cell loss in the RGC layer, endothelial dysfunction in the retinal arterioles, and an increase in reactive oxygen species (ROS) in retinal vessels [6]. Pyroptosis is defined as programmed necrosis mediated by the gasdermin family [7]. Typically, the activated inflammasome stimulates inflammatory caspases, mainly the caspase-1 family, to cleave the N-terminal fragment of gasdermin D (GSDMD-N), form GSDMD-N pores, and subsequently induce cell swelling, membrane rupture, and the release of inflammatory cytokines, finally leading to pyroptosis [8, 9]. Notably, neuroinflammation and pyroptosis are closely associated with the pathogenesis of retinal I/R injury [10]. In this work, pyroptosis of RGCs under retinal I/R conditions was further investigated.

Lysine-specific demethylase 1 (LSD1/KDM1A) is the first discovered histone demethylase that can demethylate mono- and di-methyl groups on histone H3 lysine 4 (H3K4me1/2) and histone H3 lysine 9 (H3K9me1/2) according to cellular conditions to trigger transcriptional suppression or activation [11]. Additionally, LSD1 is an important epigenetic factor to regulate inflammation and neuronal physiology [12]. Furthermore, LSD1 is extensively expressed in mouse retinal progenitor cells [13], and its inhibition possesses a neuroprotective effect on RGCs [14]. Nevertheless, whether LSD1 is involved in pyroptosis of RGCs remains to be elucidated.

MicroRNA (miRs) are a type of endogenous single-stranded noncoding RNAs with about 21 to 22 nucleotides and are involved in various biological processes, such as cell differentiation, apoptosis, embryogenesis, organogenesis, and metabolism [15, 16]. A previous review has concluded that expressions of some miRs are related to pyroptosis in I/R injury [17]. Especially, miR-21-5p participates in pyroptosis and mitochondrial dysfunction in retinal I/R injury [18], and miR-21 is regulated by demethylation of H3K4 in leukemia cells [19]. In addition, NOD-like receptor family pyrin domain-containing 12 (NLRP12), in the catalog of the NLRP family, is a protein mainly expressed in myeloid cell lineages and plays a dual role in inflammation [20]. NLRP12 is upregulated in corneal inflammatory herpetic disease induced by virulent viruses [21]. Furthermore, Chen et al. have demonstrated that NLRP12 modulates pyroptosis of RGCs in acute glaucoma through collaboration with NLRP3/NLRC4 [22]. In view of the above connections, we determined to explore the interaction between LSD1, miR-21-5p, and NLRP12 in pyroptosis of RGCs in retinal I/R injury so as to provide effective biomarkers for the treatment of ocular disorders.

## Results

### OGD/R induces pyroptosis of RGCs and upregulates LSD1

**Fig. 1:**
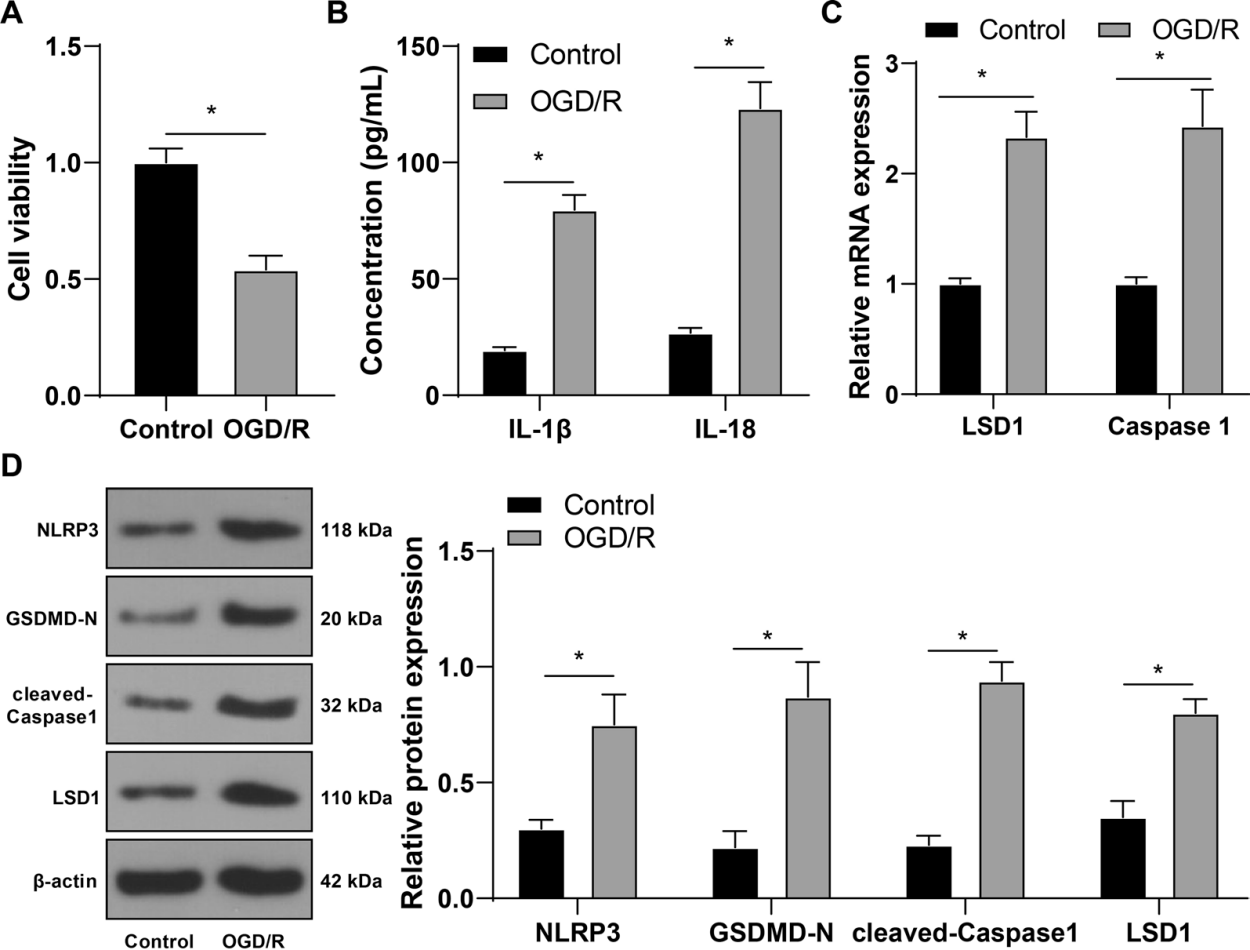
OGD/R induces pyroptosis of RGCs and upregulates LSD1. RGC-5 cells received OGD/R treatment. **A**: RGC-5 cell viability was detected via the CCK-8 method; **B**: The concentrations of IL-1β and IL-18 were examined via ELISA; **C**: The mRNA levels of LSD1 and Caspase 1 in RGC-5 cells were determined via RT-qPCR; **D**: The protein levels of NLRP3, GSDMD-N, cleaved-Caspase1, and LSD1 in RGC-5 cells were examined via Western blot analysis. Each cell experiment was performed in triplicate; measurement data were presented as mean ± standard deviation (SD); data in panel A were analyzed via the *t*-test; data in panels B-D were analyzed via two-way ANOVA, followed by Tukey’s multiple comparison test; *p < 0.05

At first, RGC-5 cells were exposed to OGD/R to mimic I/R and the changes in pyroptosis of RGC-5 cells were determined. Upon OGD/R treatment, RGC-5 cell viability was reduced (*p* < 0.05, Fig. 1A). Compared with the Control group, OGD/R treatment elevated IL-1β and IL-18 concentrations (*p* < 0.05, Fig. 1B), increased the mRNA levels of Caspase 1 (*p* < 0.05, Fig. 1C), and increased protein levels of NLRP3, GSDMD-N, and cleaved-Caspase1 (*p* < 0.05, Fig. 1D). Additionally, LSD1 can act as an anti-apoptotic agent for RGCs [14] but the expression pattern of LSD1 in OGD/R-treated RGCs is unclear. The data we obtained showed that OGD/R treatment increased LSD1 expression levels in RGC-5 cells (*p* < 0.05, Fig. 1C, D). Overall, the above results indicated that OGD/R treatment increased pyroptosis of RGCs and LSD1 expression levels.

### Silencing LSD1 represses OGD/R-induced pyroptosis of RGCs

**Fig. 2:**
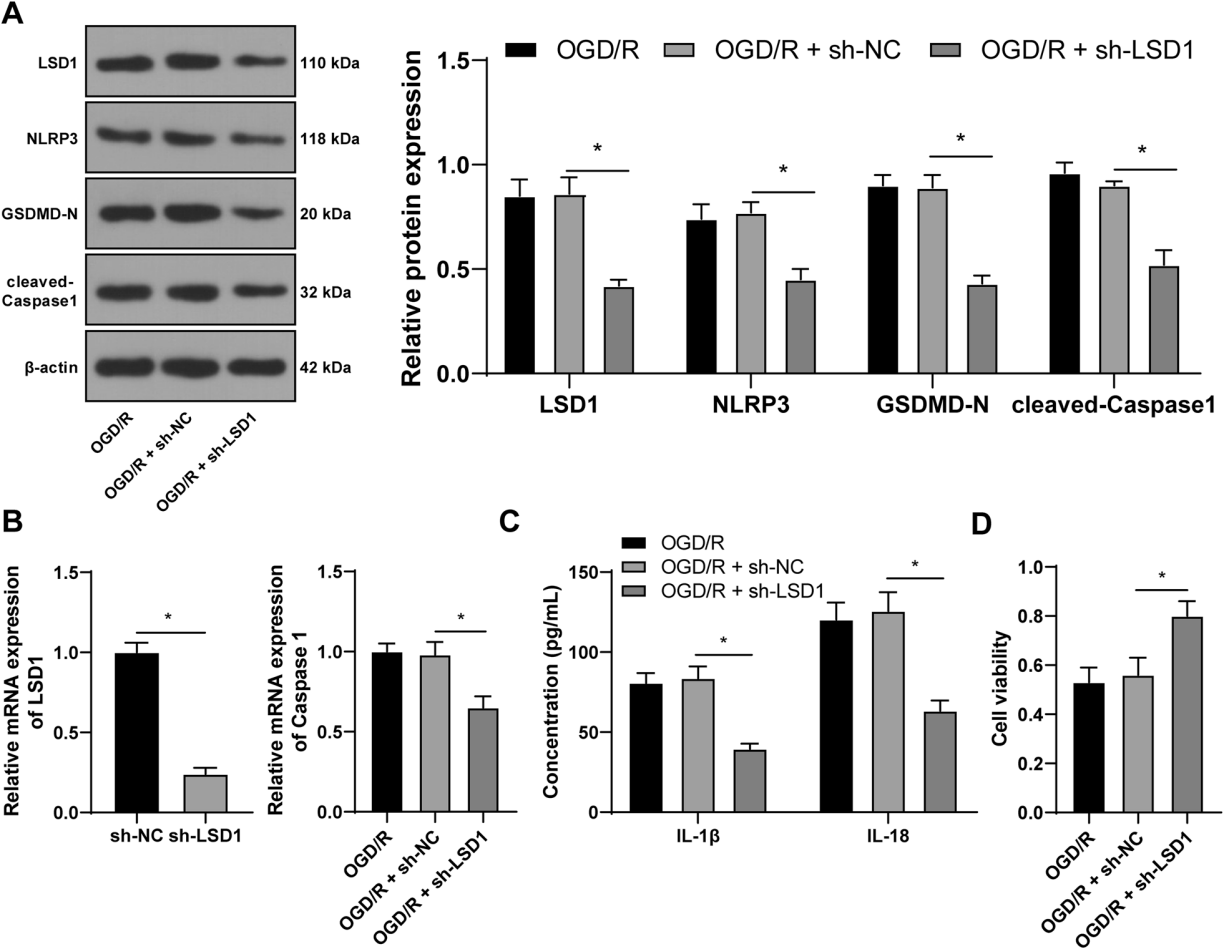
Silencing LSD1 represses OGD/R-induced pyroptosis of RGCs. RGC-5 cells were infected with shRNA-LSD1 lentivirus (sh-LSD1), with shRNA-NC lentivirus (sh-NC) as the negative control. **A**: The protein levels of LSD1, NLRP3, GSDMD-N, and cleaved-Caspase1 in RGC-5 cells were examined via Western blot analysis; **B**: The mRNA levels of LSD1 and Caspase 1 in RGC-5 cells were determined via RT-qPCR; **C**: The concentrations of IL-1β and IL-18 were examined via ELISA; **D**: RGC-5 cell viability was detected via the CCK-8 method. Each cell experiment was performed in triplicate; measurement data were presented as mean ± standard deviation (SD); data in panel B (left) were analyzed via the *t*-test; data in panels B (right) and D were analyzed via one-way ANOVA and data in panels A and C were verified via two-way ANOVA, followed by Tukey’s multiple comparison test; *p < 0.05

To clarify the role of LSD1 in OGD/R-induced pyroptosis of RGC-5 cells, we successfully suppressed LSD1 expression in RGC-5 cells through infection with shRNA-LSD1 lentivirus (sh-LSD1) (*p* < 0.05, Fig. 2A, B), and then discovered that protein levels of NLRP3, GSDMD-N, and cleaved-Caspase1 were decreased (*p* < 0.05, Fig. 2A), the mRNA levels of Caspase 1 were decreased (*p* < 0.05, Fig. 2B), and IL-1β and IL-18 concentrations were declined in OGD/R-treated RGC-5 cells (*p* < 0.05, Fig. 2C). Besides, RGC-5 cell viability in the OGD/R + sh-LSD1 group was increased compared with the OGD/R + sh-NC group (*p* < 0.05, Fig. 2D). Hence, these findings suggested that silencing LSD1 limited pyroptosis of OGD/R-treated RGCs.

### LSD1 downregulates miR-21-5p via demethylation of H3K4me2 on the miR-21-5p promoter

**Fig. 3:**
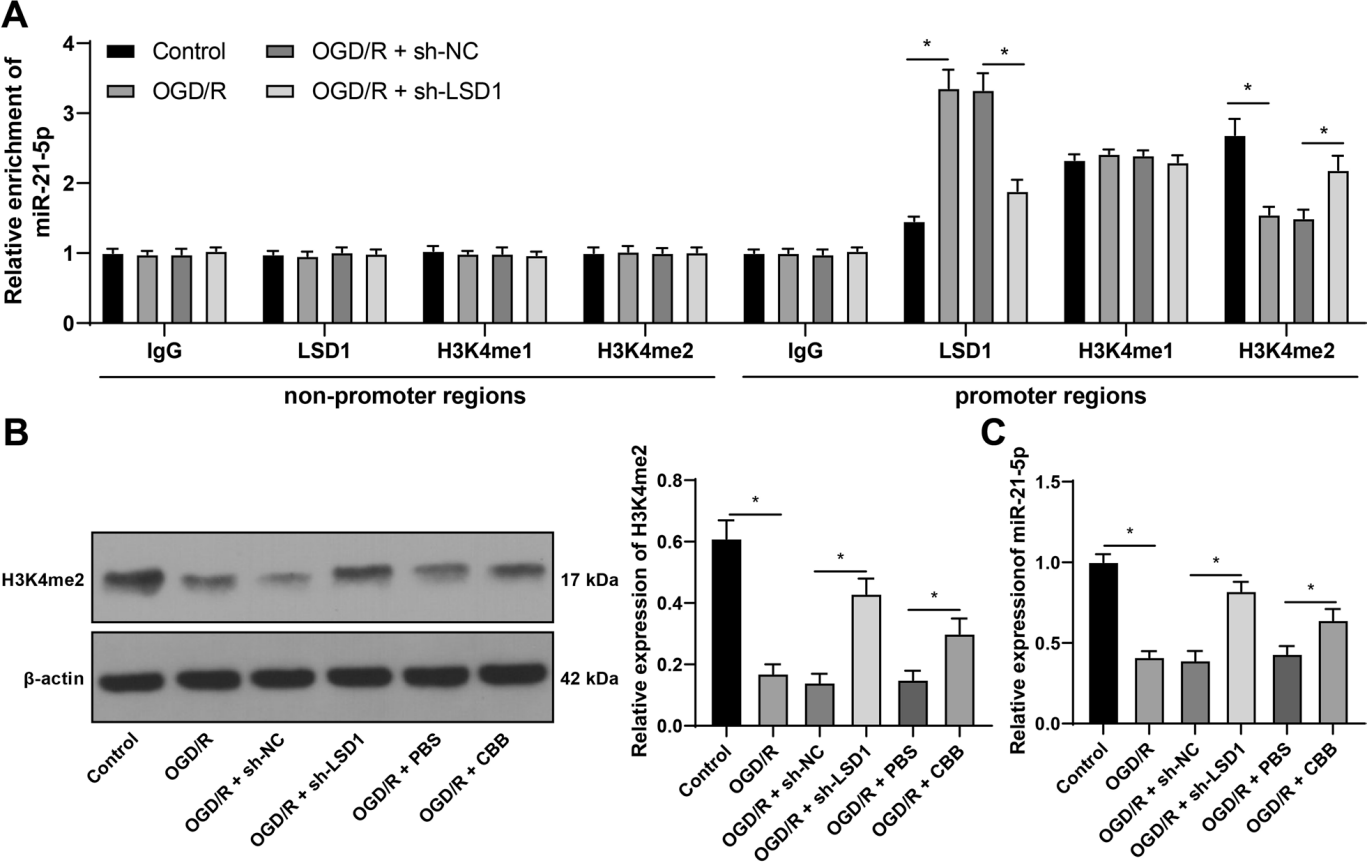
LSD1 downregulates miR-21-5p via demethylation of H3K4me2 on the miR-21-5p promoter. **A**: The relative enrichments of LSD1, H3K2me1, or H3K4me2 precipitated on the miR-21-5p promoter regions or the non-miR-21-5p promoter regions in OGD/R-treated RGC-5 cells were determined via the Ch-IP assay. CBB1007 (CBB) was the selective inhibitor of LSD1 and PBS treatment served as the control. **B**: The expression levels of H3K4me2 were examined via Western blot analysis; **C**: The expression levels of miR-21-5p in RGC-5 cells were detected via RT-qPCR. Each cell experiment was performed in triplicate; measurement data were presented as mean ± standard deviation (SD); data in panels B and C were analyzed via one-way ANOVA and data in panel A were verified via two-way ANOVA, followed by Tukey’s multiple comparison test; *p < 0.05

LSD1 suppresses the expression of downstream target genes by erasing H3K4me1/2 [23], and miR-21 is reported to be modulated by H3K4 modification [19] and miR-21-5p is under-expressed in retinal I/R [18]. Therefore, miR-21-5p may be a potential target of LSD1. The Ch-IP assay showed that, after OGD/R treatment, LSD1 enrichment on the miR-21-5p promoter was increased and H3K4me2 enrichment on the miR-21-5p promoter was reduced, while sh-LSD1 treatment showed the opposite outcomes (*p* < 0.05, Fig. 3A). However, H3K4me1 enrichment on the miR-21-5p promoter had no significant change (Fig. 3A). Meanwhile, OGD/R treatment decreased H3K4me2 expression level whilst sh-LSD1 increased H3K4me2 expression level (*p* < 0.05, Fig. 3B). Furthermore, miR-21-5p expression levels in the OGD/R group were decreased compared with the Control group, while miR-21-5p expression levels in the OGD/R + sh-LSD1 group were increased compared with the OGD/R + sh-NC group (*p* < 0.05, Fig. 3C). To further verify whether LSD1 affected miR-21-5p expression through H3K4me2, H3K4me2 expression in RGC-5 cells was elevated using CBB1007 (CBB) (*p* < 0.05, Fig. 3B), after which miR-21-5p expression levels were increased (*p* < 0.05, Fig. 3C). By and large, these above findings indicated that LSD1 can induce demethylation of H3K4me2 on the miR-21-5p promoter to repress miR-21-5p expression.

### Silencing miR-21-5p averts the repressive effect of silencing LSD1 on OGD/R-induced pyroptosis of RGCs

**Fig. 4:**
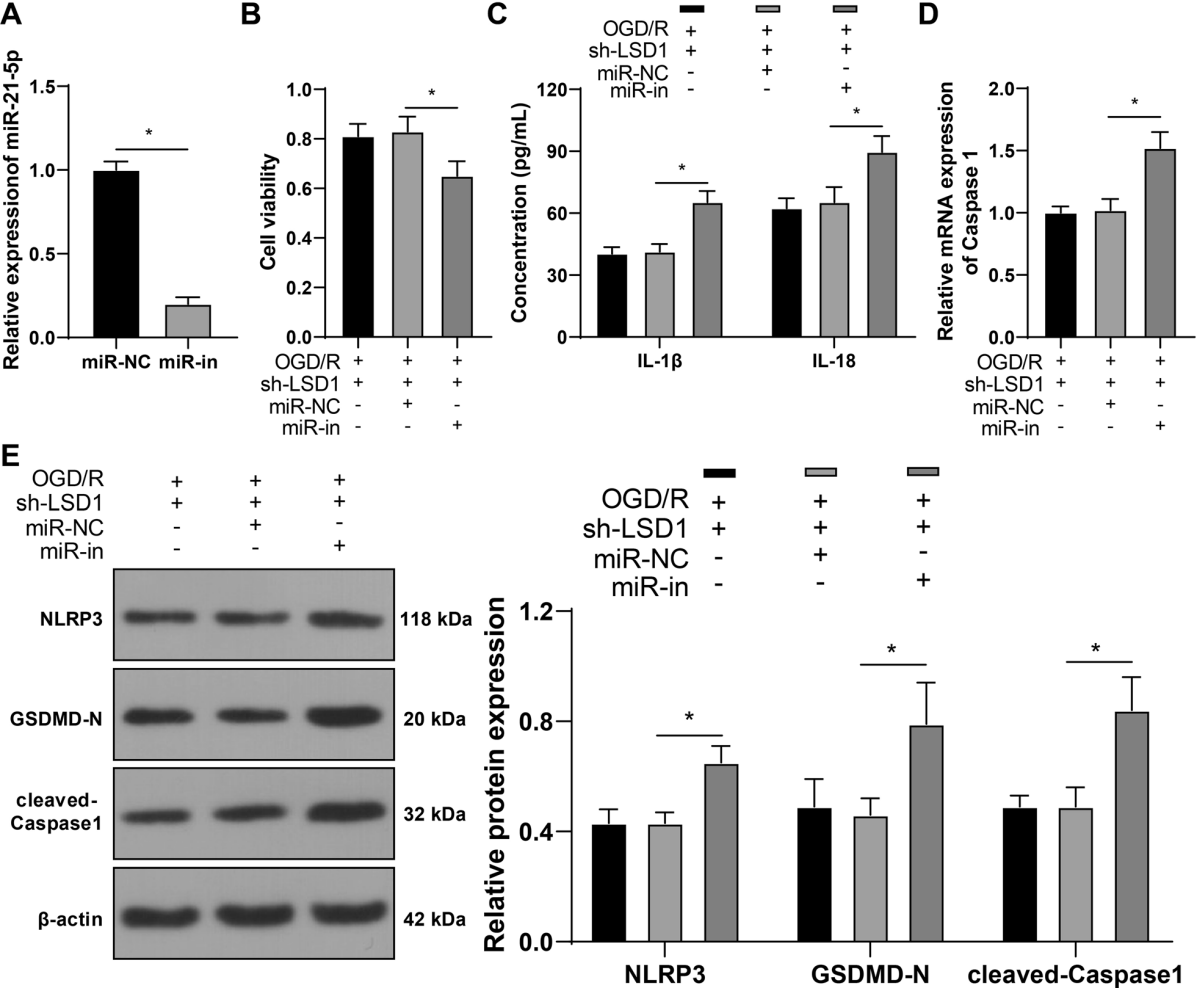
Silencing miR-21-5p averts the repressive effect of silencing LSD1 on OGD/R-induced pyroptosis of RGCs. RGC-5 cells were infected with miR-21-5p inhibitor lentivirus (miR-in), and inhibitor NC lentivirus (miR-NC) was used as the negative control. **A**: The expression levels of miR-21-5p in RGC-5 cells were examined via RT-qPCR; **B**: RGC-5 cell viability was detected via the CCK-8 method; **C**: The concentrations of IL-1β and IL-18 were examined via ELISA; **D**: The mRNA levels of Caspase 1 in RGC-5 cells were determined via RT-qPCR; **E**: The protein levels of NLRP3, GSDMD-N, and cleaved-Caspase1 in RGC-5 cells were examined via Western blot analysis. Each cell experiment was performed in triplicate; measurement data were presented as mean ± standard deviation (SD); data in panel A were analyzed via the *t*-test; data in panels B and D were analyzed via one-way ANOVA and data in panels C and E were analyzed via two-way ANOVA, followed by Tukey’s multiple comparison test; *p < 0.05

Subsequently, RGC-5 cells were infected with miR-21-5p inhibitor lentivirus (miR-in) to suppress the intracellular expression of miR-21-5p (*p* < 0.05, Fig. 4A) and combined with sh-LSD1. Thereafter, testing results showed that miR-21-5p knockdown reduced RGC-5 cell viability (*p* < 0.05, Fig. 4B) and increased IL-1β and IL-18 concentrations (*p* < 0.05, Fig. 4C), the mRNA levels of Caspase 1 (*p* < 0.05, Fig. 4D), and the protein levels of NLRP3, GSDMD-N, and cleaved-Caspase1(*p* < 0.05, Fig. 4E). Altogether, silencing miR-21-5p neutralized the effect of LSD1 suppression on OGD/R-induced pyroptosis of RGCs.

### miR-21-5p targets and binds to NLRP12 to downregulate NLRP12

**Fig. 5:**
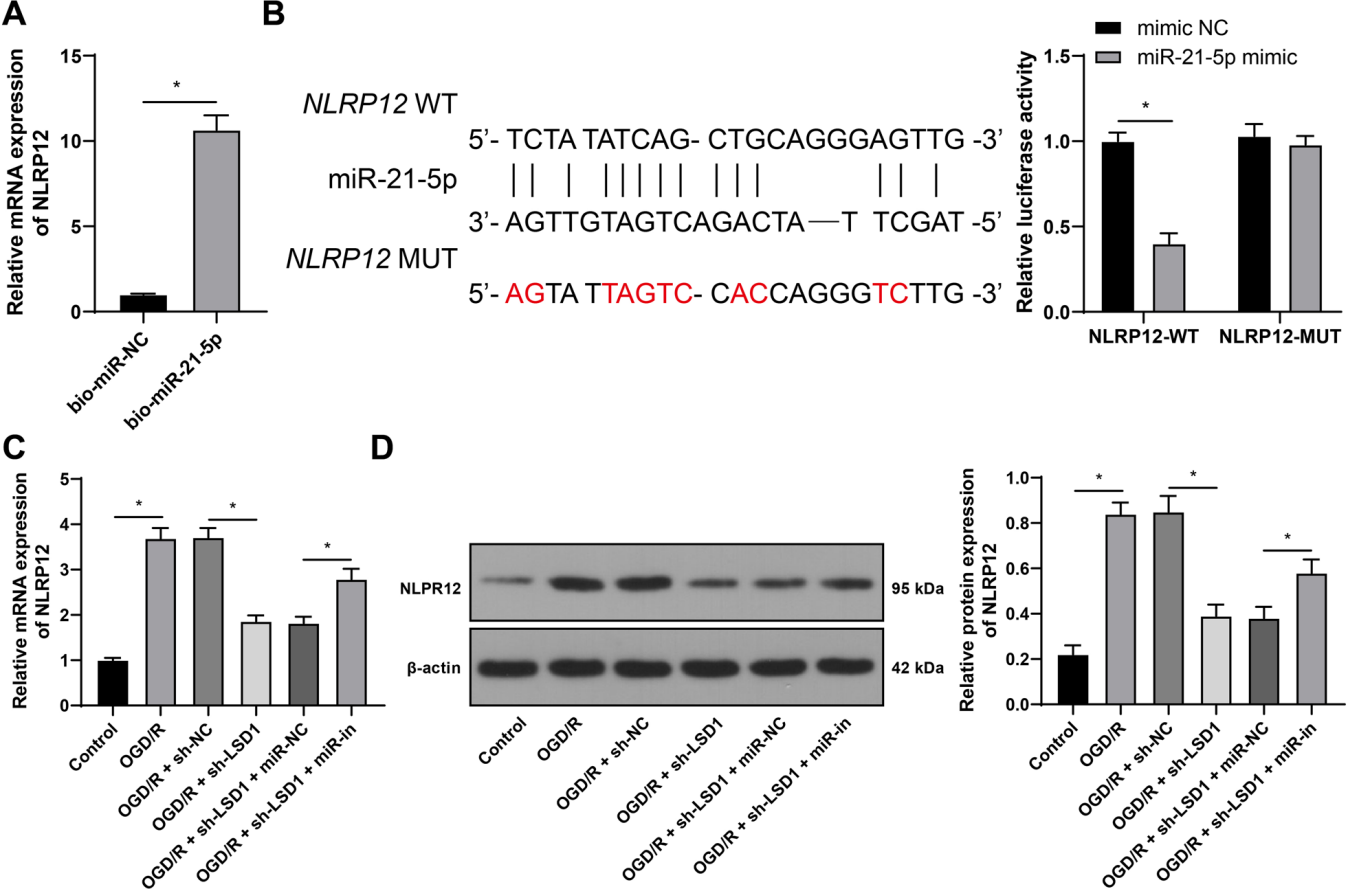
miR-21-5p targets and binds to NLRP12 to downregulate NLRP12. **A**-**B**: The binding of miR-21-5p to NLRP12 was detected via RNA pull-down and dual-luciferase assays; **C**-**D**: The expression levels of NLRP12 were examined via RT-qPCR and Western blot analysis. Each cell experiment was performed in triplicate; measurement data were presented as mean ± standard deviation (SD); data in panel A were analyzed via the *t*-test; data in panels C and D were analyzed via one-way ANOVA and data in panel B were analyzed via two-way ANOVA, followed by Tukey’s multiple comparison test; *p < 0.05

Next, after predicting the downstream target genes of miR-21-5p using the RNA22 v2 database, we focused on NLRP12. NLRP12 is involved in pyroptosis of ganglion cells in acute glaucoma [22]. RNA pull-down assay revealed that, compared with bio-miR-NC, bio-miR-21-5p could pull down more NLRP12 (*p* < 0.05, Fig. 5A). Based on the obtained binding sites, the dual-luciferase assay was designed and carried out, which disclosed the binding relationship between miR-21-5p and NLRP12 (*p* < 0.05, Fig. 5B). Besides, OGD/R treatment increased NLRP12 expression levels, while sh-LSD1 treatment suppressed NLRP12 expression levels and this suppression was partially counteracted by miR-in treatment (*p* < 0.05, Fig. 5C, D). Collectively, miR-21-5p downregulated NLRP12 through targeting and binding to NLRP12.

### NLRP12 overexpression averts the repressive effect of silencing LSD1 on OGD/R-induced pyroptosis of RGCs

**Fig. 6:**
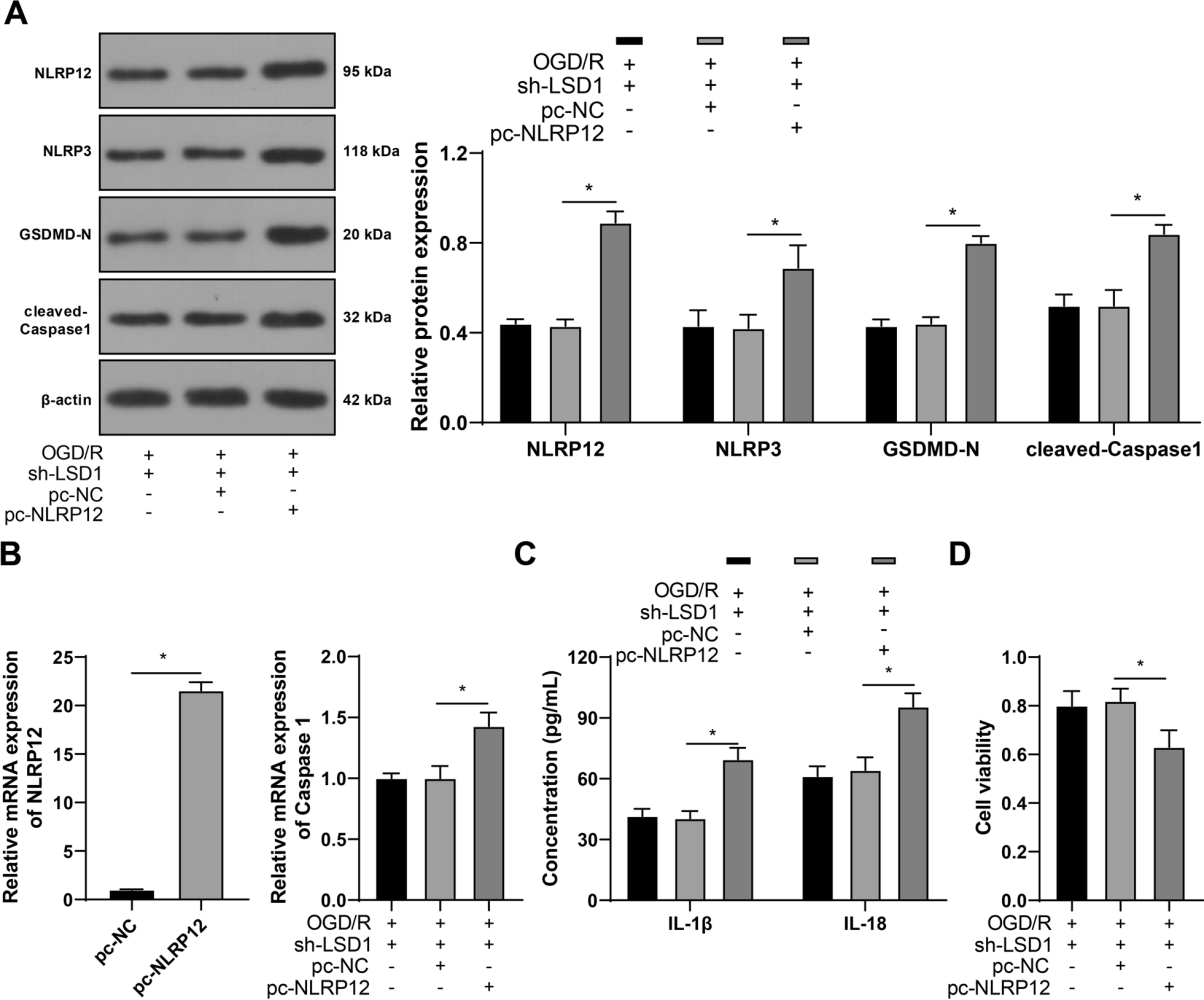
NLRP12 overexpression averts the repressive effect of silencing LSD1 on OGD/R-induced pyroptosis of RGCs. RGC-5 cells were infected with pcDNA3.1-NLRP12 lentivirus (pc-NLRP12), with pcDNA3.1-NC lentivirus (pc-NLRP12) as the negative control. **A**: The protein expressions of NLRP12, NLRP3, GSDMD-N, and cleaved-Caspase1 in RGC-5 cells were examined via Western blot analysis; **B**: mRNA expressions of NLRP12 and Caspase 1 in RGC-5 cells determined via RT-qPCR; **C**: concentrations of IL-1β and IL-18 examined via ELISA; **D**: RGC-5 cell viability was detected via the CCK-8 method. Each cell experiment was performed in triplicate; measurement data were presented as mean ± standard deviation (SD); data in panel B (left) were analyzed via the *t*-test; data in panels B (right) and D were analyzed via one-way ANOVA and data in panels A and C were analyzed via two-way ANOVA, followed by Tukey’s multiple comparison test; *p < 0.05

At last, RGC-5 cells were infected with pcDNA3.1-NLRP12 lentivirus (pc-NLRP12) to increase NLRP12 expression (*p* < 0.05, Fig. 6A, B) and combined with sh-LSD1. Through detections, it was found that NLRP12 increase elevated NLRP3, GSDMD-N, and cleaved-Caspase1 protein expressions (*p* < 0.05, Fig. 6A), Caspase 1 mRNA levels (*p* < 0.05, Fig. 6B), and IL-1β and IL-18 concentrations (*p* < 0.05, Fig. 6C), while reduced RGC-5 cell viability (*p* < 0.05, Fig. 6D). Overall, the above results suggested that NLRP12 overexpression counteracted the effect of silencing LSD1 on OGD/R-induced pyroptosis of RGCs.

## Discussion

AS the sole output element and projection neuron of the retina, RGC degeneration stands as a hallmark of many optic neuropathies that affects millions of people throughout the world [24]. Besides, during retinogenesis, histone methylation is proven to participate in retinal homeostasis and differentiation by affecting cell lineage-specific mechanisms [25]. In this present work, we revealed that LSD1 facilitated OGD/R-induced RGC pyroptosis via modulating the miR-21-5p/NLRP12 axis.

Previous studies have illustrated that pyroptosis induces RGC death and further affects the pathogenesis of ocular diseases [26, 27]. Additionally, OGD/R treatment is often applied to mimic I/R-related neuronal injury models and is demonstrated to induce neuronal pyroptosis, apoptosis, and inflammation [10, 28, 29]. With reference to the properties of OGD/R treatment, RGC-5 cells were subjected to OGD/R exposure. As expected, OGD/R exposure reduced RGC cell viability and boosted inflammation and pyroptosis. The results of a relevant study have elucidated that OGD/R exposure triggers pyroptosis of rat retinal precursor R28 cells with the elevation of intracellular NLRP3, GSDMD-N, and proinflammatory cytokines [30]. Hence, the above findings confirmed that OGD/R exposure induced RGC pyroptosis.

LSD1 inhibitors are regarded as drug candidates for the treatments of neurodegeneration, viral diseases, and cancer [31]. Tranylcypromine-mediated LSD1 suppression limits death and promotes cell survival in glutamate-induced RGCs [14], and LSD1 inhibition maintains rod photoreceptors and supports retinal functions in retinitis pigmentosa [32]. Our experiments revealed that LSD1 expression levels in RGC-5 cells were elevated after OGD/R exposure and silencing LSD1 increased RGC-5 cell viability and declined the levels of pyroptosis-related factors and proinflammatory cytokines. Previous studies have illustrated that LSD1 triggers and intensifies the inflammatory response via crosstalk with PKCα/nuclear factor κB [33] and LSD1 suppression motivates autophagy and further represses oxidized low-density lipoprotein-induced NLRP3 activation and inflammatory responses [34], making it plausible that LSD1 suppression limited OGD/R-induced pyroptosis of RGCs.

Furthermore, LSD1 inhibition possesses a positive role in modulating the Nrf2-Gclc-glutathione cascade via increasing H3K4me1/2 and mitigates diabetic retinopathy [35]. Interestingly, KDM5A, another histone demethylase, is previously reported to downregulate miR-21 expression via demethylating H3K4me3 located in the miR-21 locus [19]. Our subsequent experiments confirmed the binding of LSD1 to miR-21-5p, and that LSD1 downregulated miR-21-5p via demethylation of H3K4me2 on the miR-21-5p promoter. Besides, through literature review, we found that exosomal-miR-21 maintains photoreceptor viability and ameliorates retinal degeneration [36], and miR-21 knockdown abrogates the anti-pyroptotic role of interleukin-35 in retinal I/R injury [10]. Akin to miR-21, miR-21-5p overexpression in gingival mesenchymal stem cells-derived exosomes promotes RGC survival and hampers neuroinflammation in acute glaucoma induced by retinal I/R injury [37] and the lncRNA H19/miR-21-5p/PDCD4 network participates in microglial pyroptosis and death after retinal I/R injury [18] Thereafter, RGC-5 cells underwent a rescue experiment with miR-21-5p inhibitor and sh-LSD1. Notably, silencing miR-21-5p facilitated inflammation and pyroptosis in RGC-5 cells. Altogether, our findings and existing evidence proved that silencing miR-21-5p rescued LSD1 inhibition-mediated limitation of RGC pyroptosis induced by OGD/R.

Previous studies have documented that silencing NLRP10 attenuates neuroinflammation in I/R-associated brain injury via inactivating the NLRP12/ASC/Caspase-1 pathway [38], and NLRP12/NLRC4 is responsible for GSDMD-triggered pyroptosis in dry eye disease [39]. In follow-up prediction and detection, the binding of miR-21-5p to NLRP12 was validated, and silencing LSD1 decreased NLRP12 expression whilst miR-21-5p overexpression promoted NLRP12 expression. Finally, RGC-5 cells overexpressing NLRP12 were merged with sh-LSD1, after which RGC-5 cell viability was reduced and pyroptosis was increased. Consistently, a preceding study has elucidated that NLRP12 activation stimulates retinal I/R injury, and the suppression of NLRP12/NLRP3/NLRC4 rescues RGC loss and retina injury via limiting neuroinflammation and pyroptosis [22]. Altogether, these results elicited that NLRP12 overexpression averted LSD1 suppression-repressed pyroptosis of OGD/R-treated RGCs.

## Conclusions

**Fig. 7:**
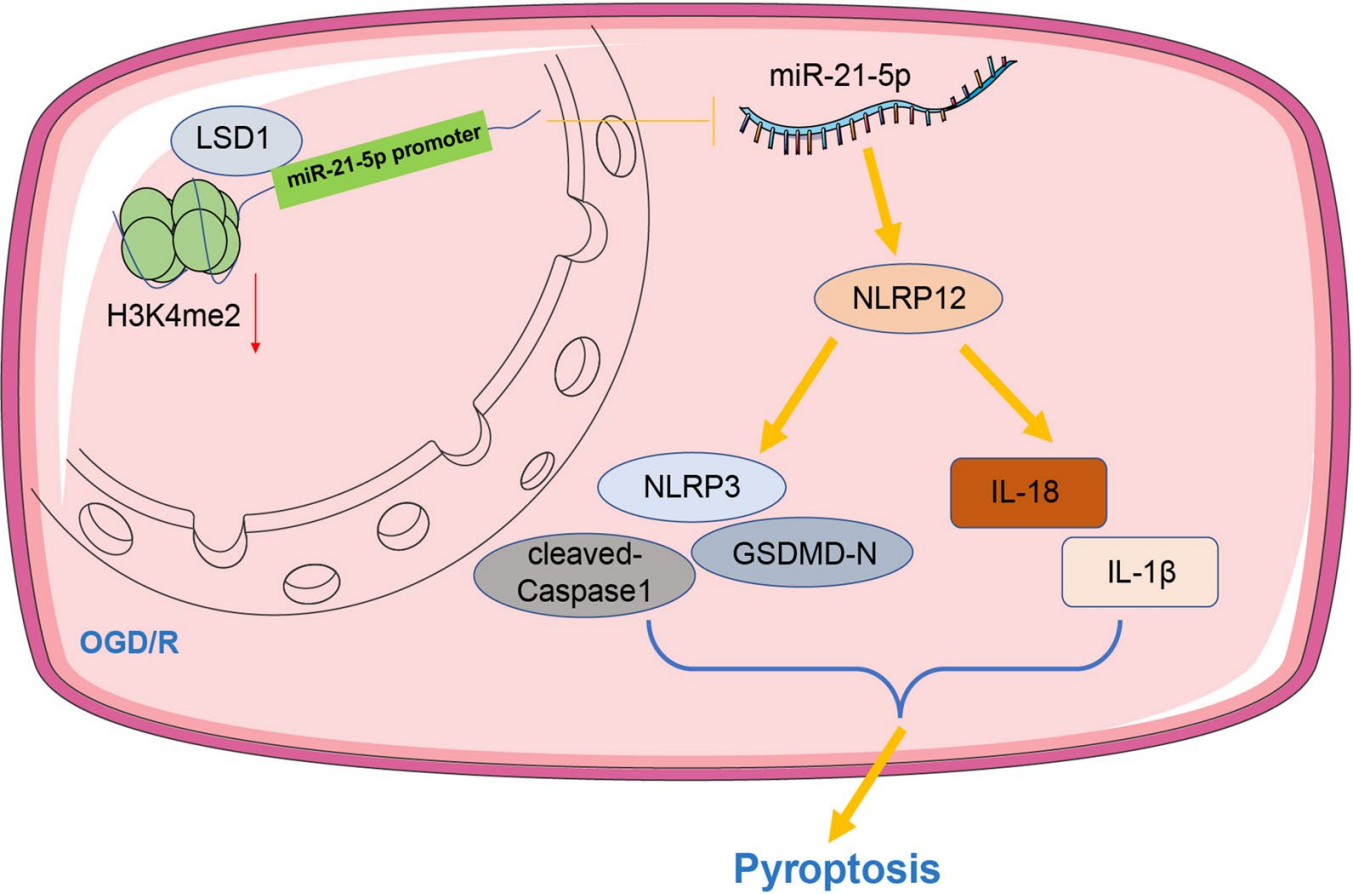
Mechanism diagram. The molecular mechanism of LSD1 in OGD/R-induced pyroptosis of RGCs. LSD1 downregulated miR-21-5p via demethylating H3K4me2 on the miR-21-5p promoter, which inhibited the binding of miR-21-5p to NLRP12 and further upregulated NLRP12, finally promoting pyroptosis of OGD/R-induced RGCs

All in all, our data preliminarily and firstly expounded that LSD1 decreased miR-21-5p expression via demethylating H3K4me2 on the miR-21-5p promoter to upregulate NLRP12 expression, boosting OGD/R-induced pyroptosis of RGCs (Fig. 7). This work is expected to provide feasible strategies for attenuating pyroptosis of RGCs induced by retinal I/R injury and suggested that the LSD1/miR-21-5p/NLRP12 mechanism may provide promising therapeutic ways for ocular disorders. Nevertheless, we only verified this mechanism at the cell level and failed to investigate whether LSD1 affects other aspects of retinal I/R injury or to explore other potential downstream mechanisms associated with LSD1 and miR-21-5p. Moving forward, we shall explore the underlying effects of LSD1 on the pathology of retinal I/R injury in animal models and probe other practicable downstream mechanisms of LSD1 or miR-21-5p.

## Methods

### Cell culture and treatment

Retinal ganglion cells (RGC-5, BNCC341515; BeNa Culture Collection, Beijing, China) were inoculated on the Dulbecco’s modified Eagle’s medium (DMEM; Gibco, Grand Island, NY, USA) with a combination of 10% fetal calf serum, 100 U/mL penicillin, and 100 μg/mL streptomycin (Gibco) at 37 ℃ with 5% CO_2_. Thereafter, the medium was changed with a sugar-free DMEM, and RGC-5 cells were cultured in a hypoxic incubator for 3 h to simulate the OGD milieu, after which the sugar-free DMEM was replaced with a normal DMEM, and the culture plates were routinely cultured in a saturated oxygen incubator for 12 h to established the OGD/R models [18]. The control cells were not exposed to OGD/R. Lentiviruses (GeneChem, Shanghai, China), including shRNA-LSD1 lentivirus, shRNA-NC lentivirus, pcDNA3.1-NLRP12 lentivirus, and pcDNA3.1-NC lentivirus, miR-21-5p inhibitor, and inhibitor NC lentiviruses, were used to infect RGC-5 cells for 6–8 h at multiplicity of infection of 30. Afterwards, the stably expressed RGC-5 cells were selected using puromycin (5 μg/mL; Solarbio, Beijing, China) and then subjected to OGD/R treatment. CBB1007 (MedChemExpress, Monmouth Junction, NJ, USA) was a selective inhibitor of LSD1, with the half maximal inhibitory concentration of 5.27 nM, and was treated with phosphate-buffered saline (PBS) as the control.

### Cell counting kit-8 (CCK-8) method

RGC-5 cells (100 μL; 3 × 10^3^ cells/well) were seeded in 96-well plates and maintained at 37 °C with 5% CO_2_ for 12 h. After 24 h of experimental treatment, each well of the plates was added with 10 μL CCK-8 reagent (KTC011001; Abbkine, Wuhan, Hubei, China) and incubated for 2 h. Thereafter, the optical density at 450 nm was determined using a microplate reader (Infinite M200; Tecan, Melbourne, Victoria, Austria) to calculate RGC-5 cell viability.

### Enzyme-linked immunosorbent assay (ELISA)

RGC-5 cell culture supernatant underwent centrifugation at 1000 g for 20 min to eliminate the precipitate, and the concentration of protein was examined using the bicinchoninic acid protein assay kit (Beyotime, Shanghai, China). Then, the contents of interleukin (IL)-18 and IL-1β in 100 μL of the supernatant were determined using IL-18 ELISA kit (ab216165; Abcam, Cambridge, MA, USA) and IL-1β kit (ab197742; Abcam) based on the producer’s instructions.

### Chromatin immunoprecipitation (Ch-IP) assay

We conducted the Ch-IP assay according to the protocols of Millipore Ch-IP (Billerica, MA, USA). RGC-5 cells were cross-linked in formaldehyde solution at 37 °C for 10 min and subjected to ultrasonic treatment to obtain chromatin fragments, and then the immunoprecipitation reaction on these chromatin fragments was conducted using the antibodies against LSD1 (ab129195; Abcam), H3K4me1 (ab176877; Abcam), H3K4me2 (ab32356; Abcam), and IgG (ab133470, Abcam), from which DNA was then obtained and purified for PCR amplification. The PCR primers of the miR-21 promoter: 5′-TGTCGTGGTCGTGACATCGCAT-3′ (forward) and 5′-CCTACCAGGCAAAACAAAATG-3′ (reverse). The PCR primers of the non-miR-21 promoter: 5′-CCACCTTGTCGGATAGCTTATC-3′ (forward) and 5′-CAAAATGTCAGACAGCCCATCG-3′ (reverse).

### Dual-luciferase assay

NLRP12 mRNA containing binding sites to miR-21-5p was cloned into the pGL3 luciferase vector (Promega, Madison, WI, USA) to construct the plasmid NLRP12-wild-type (NLRP12-WT), and similarly, NLRP12 mRNA containing mutated binding sites to miR-21-5p was cloned into pGL3 vector to construct the plasmid NLRP12 mutant-type (NLRP12-MUT), and then these plasmids were transfected into RGC-5 cells with miR-21-5p mimic or the negative control using Lipofectamine 3000. Based on the manufacturer’s requirements, the luciferase activity was measured using a dual-luciferase reporter gene assay kit (Promega) 48 h post transfection.

### RNA pull-down assay

RGC-5 cell lysates were obtained using the IP lysis buffer (Beyotime) and incubated with biotinylated miR-21-5p or miR-NC probes (bio-miR-21-5p or bio-miR-NC), followed by incubation with M-280 streptavidin magnetic beads (100 µL; Sigma-Aldrich, St. Louis, MO, USA) for 4 h at 4 °C. Then, the magnetic beads were precipitated using a magnetic bar. Afterwards, the samples were rinsed 4 times with the lysis buffer and purified using the TRIzol reagent (Invitrogen, Carlsbad, CA, USA). And, the level of NLRP12 obtained from precipitation was detected using real-time quantitative polymerase chain reaction (RT-qPCR).

### RT-qPCR

The total RNA of RGC-5 cells was isolated and the total RNA (1 μg) was reverse-transcribed into the complementary DNA using a Sprint R-T Complete-Double PrePrimed kit (Clontech, Mountain View, CA, USA). RT-qPCR reactions were conducted on SYBR Green qPCR Premix (Clontech) and ABI 7500 system (Applied Biosystems, Foster City, CA, USA). With GAPDH or U6 [40] as the internal reference, the relative expressions of genes were processed using the 2^−△△Ct^ method, and the PCR primers are exhibited in Table 1.

**Table 1:** qPCR primers

Gene	Primers (5′-3′)
*LSD1*	F: GATGAAAGCTTGGCCAACCTCT
	R: AAGTGAGCCGGAAGAGCCGTCT
*miR-21-5p*	F: GCGTCGGTAGCTTATCAGACT
	R: CTCAACTGGTGTCGTGGAGTC
*NLRP12*	F: CCTGGGGACGAATGGAGAAG
	R: CTGAAAGGTGCTGAGAGCCA
Caspase 1	F: GGACCCTCAAGTTTTGCCCT
	R: AGACGTGTACGAGTGGTTGT
GAPDH	F: GGTCCCAGCTTAGGTTCATCA
	R: AATCCGTTCACACCGACCTT
U6	F: CGCTTCGGCAGCACATATACT
	R: CTTCACGAATTTGCGTGTCAT

### Western blotting

RGC-5 cells were lysed using the radioimmunoprecipitation assay lysis buffer supplemented with protease inhibitor (Beyotime). The total protein in cells was resolved using sodium dodecyl sulfated-polyacrylamide gel electrophoresis and shifted to polyvinylidene difluoride membranes, after which the membranes were blocked using 5% skim milk for 1 h and incubated with antibodies against NLRP3 (ab263899, 1:1000, Abcam), GSDMD-N (MBS9629047, 1:1000, MyBioSource, San Diego, CA, USA), cleaved-Caspase1 (PA5-38,099, 1:500, ThermoFisher, Waltham, MA, USA), LSD1 (ab129195, 1:10,000, Abcam), NLRP12 (PA5-21,027, 1 µg/mL, ThermoFisher), H3K4me2 (ab32356, 1:2000, Abcam), and β-actin (ab8227, 1:1000, Abcam) overnight at 4 °C, and then incubated with secondary antibody (1:2000, ab205718, Abcam) for 1 h. The visualization of protein blots was achieved using the enhanced-chemiluminescence Plus Western Blot analysis system, and the grayscale values were evaluated using Quantity One (MP4000; Bio-Rad, Hercules, CA, USA) to reflect the relative levels.

### Statistical analysis

SPSS 21.0 statistical software (IBM SPSS Statistics, Chicago, IL, USA) and GraphPad Prism 8.0 software were employed for data analyses and graphing. Firstly, experimental data were verified to comply with normality and homogeneity of variance. Then, the *t*-test was adopted for comparisons between 2 groups, and one-way analysis of variance (ANOVA) or two-way ANOVA followed by Tukey’s post-hoc was adopted for comparisons among multiple groups. *p* < 0.05 indicated statistical significance.

## Data Availability

All data generated in this published article can be available with corresponding author.
